# The effects of two vitamin D regimens on ulcerative colitis activity index, quality of life and oxidant/anti-oxidant status

**DOI:** 10.1186/s12937-019-0441-7

**Published:** 2019-03-11

**Authors:** Sara Karimi, Sanam Tabataba-vakili, Zahra Yari, Forough Alborzi, Mehdi Hedayati, Nasser Ebrahimi-Daryani, Azita Hekmatdoost

**Affiliations:** 1 Department of Clinical Nutrition and Dietetics, Faculty of Nutrition Sciences and Food Technology, National Nutrition and Food Technology, Research Institute, Shahid Beheshti University of Medical Sciences, Tehran, Iran; 2 Department of Medicine, Shahid Beheshti University of Medical Sciences, Tehran, Iran; 3 Division of Gastroenterology, Imam Khomeini Hospital, School of Medicine, Tehran University of Medical Sciences, Tehran, Iran; 4 Cellular and Molecular Endocrine Research Center, Research Institute for Endocrine Sciences, Shahid Beheshti University of Medical Sciences, Tehran, Iran

**Keywords:** Ulcerative colitis, Inflammatory bowel disease, Vitamin D, Oxidative stress

## Abstract

**Background:**

The optimum dosage for vitamin D supplementation has not yet been elucidated in patients with Ulcerative colitis (UC). The aim of this study was to investigate the effects of two vitamin D regimens in UC patients with vitamin D deficiency.

**Methods:**

In this double blind randomized clinical trial, 50 patients with mild to moderate UC, who met inclusion criteria, received either 1000 or 2000 IU/day of vitamin D (as low dose or high dose group, respectively) for 12 weeks. Serum 25-hydroxy vitamin D (25-OHD) level, total antioxidant capacity (TAC), and Total Oxidant Status (TOS), the inflammatory bowel disease questionnaire − 9 (IBDQ-9) score and the Simple Clinical Colitis Activity Index Questionnaire (SCCAI) score were assessed before and after intervention.

**Results:**

At the end of study, serum 25-OHD levels significantly increased in the high dose group (*P* < 0.001) and the increase was significantly more than low dose group (6.7 ± 3.8 ng/mL in the high dose group versus 0.2 ± 0.5 ng/mL in the low dose group) (*P* < 0.001). Serum TOS concentration decreased significantly (− 0.37 ± 0.26) only in the high dose group (*P* value = 0.023). There was no statistically significant change in serum TAC between two groups during the study. IBDQ-9 mean score significantly increased in high dose group compared to the low dose group (*P* value = 0.001) and SCCAI score in both groups reduced (− 2.58 ± 2.16 and − 0.9 ± 0.3 in high dose and low dose respectively), while this reduction was significant only in the high dose group (*P* value ≥0.001).

**Conclusion:**

Our results indicate that 2000 IU daily dose of vitamin D can increase serum 25-OHD concentration, and quality of life, while it reduces disease activity in UC patients with vitamin D deficiency. We recommend assessment of the vitamin D status in all patients with UC because they may benefit from vitamin D therapy.

## Introduction

Inflammatory bowel disease (IBD) is a type of immune-mediated chronic bowel disorder including Ulcerative colitis (UC) and Crohn’s disease (CD) [1]. The etiology of IBD is not completely understood; however, increasing evidence have shown the role of genetic and environmental factors on immunopathologic processes of disease [2–8].

The incidence and prevalence of IBD is increasing over time in western countries and in different parts of the world [9]. The prevalence of UC in Europe and North America has been reported to be 505 and 249 out of every 100,000 persons, respectively [10]. Although little epidemiological information of developing countries is available, recent studies indicate that this disease is rapidly increasing in many developing countries as well as Africa, South America and Asia [9–11]. In spite of significant advances in the treatment of this disease, no definitive treatment has yet been found so far and the aim of existing treatments is to reduce symptoms, prolongation of disease remission and improvement in patients’ quality of life. These medications have serious side effects such as increasing the risk of infection, increasing the sensitivity and risk of mutagenesis, which limits their therapeutic value [1, 12–14].

Vitamin D has been linked to a wide range of physiological functions including immune responses [15]. Vitamin D deficiency has been associated with various immunological diseases such as allergies and autoimmune diseases. Different mechanisms for the effects of vitamin D on inherent and acquired immune systems are supposed to reduce inflammation, promote immunological tolerance, and increase the intestinal epithelial integrity [16].

Several studies have been conducted to evaluate the efficacy of vitamin D in IBD patients and in some of them a link between vitamin D deficiency and disease activity, mortality and severity of the disease, its early onset and risk of recurrence was found [17–22]; however, the optimum dosage for supplementation has not yet been elucidated. Thus, we designed this study to determine the effects of two dosages of vitamin D supplementation on serum vitamin D, total antioxidant capacity (TAC), total oxidant status (TOS), quality of life, and disease activity index in patients with UC.

## Materials and methods

### Study design

**Fig. 1:**
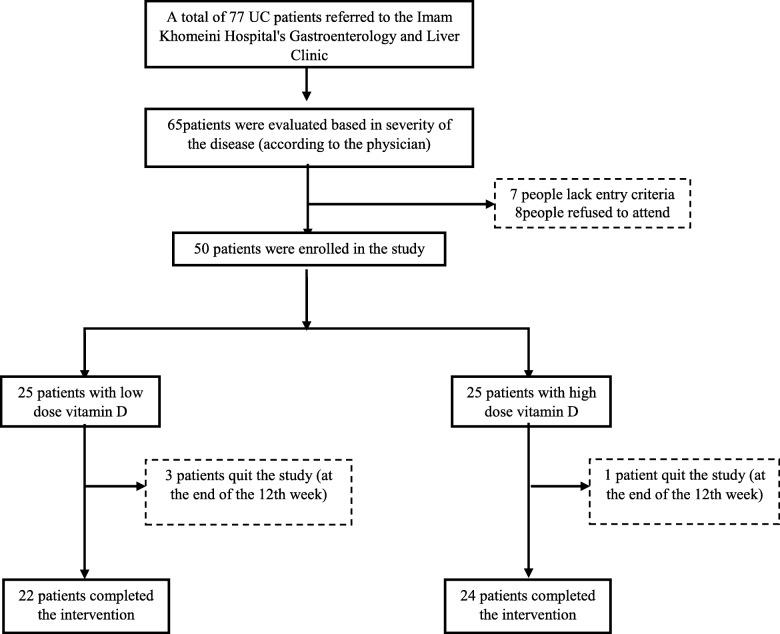
Patients’ recruitment flow chart

This study is a double blind randomized clinical trial, which included patients with mild to moderate UC [14] referring to Shahid Fayyaz-Bakhsh Hospital, and a private gastroenterology clinic, who fulfilled the inclusion criteria of the study. At the beginning of the study, the goals and methods of the study were explained to patients. Out of 77 patients, 65 UC patients were interviewed and 50 patients were willing to take part in our study (Fig. 1). Written consent approved by the ethics committee of the National Nutrition and Food Technology Research Institute (NNFTRI), Shahid Beheshti University of Medical Sciences (SBMU), Tehran, Iran, was obtained from all patients. A general demographic questionnaire was completed for each patient. Meanwhile, the inflammatory bowel disease questionnaire-9 (IBDQ-9) and the Simple Clinical Colitis Activity Index Questionnaire (SCCAIQ) were completed. [14]. The IBDQ-9 questionnaire was designed by Casellas et al. [23]. To measure the quality of life affected by IBD from the original IBD questionnaire. This questionnaire contains 9 questions, which like the original version, assesses the condition from the four dimensions of gastrointestinal, systemic, emotional and social disturbances. The answer to each question has 7 choices that range from 1 (the worst) to 7 (the best). The patient should mark one of the options as the best answer for each question, and the total score is 9 to 63. The higher score represents a better quality of life in patients. It is worth noting that this questionnaire has been linguistically validated for Iranian patients [24]. SCCAI-Q which is suitable for evaluation of patients with UC is a clinical activity indicator and consists of 6 questions, scaled from zero to 18.

In order to evaluate patients’ dietary intakes, three 24-h food recalls on one holiday and two working days were completed through telephone or in-person interviews at the beginning and end of study. Patients were asked not to change their diet and physical activity during the study period. The analysis of 24-h food recall questionnaires was done using Nutritionist IV (N4) (First Databank, Hearst Corp, San Bruno, CA, USA).

### Ethical considerations

This study was conducted in accordance with principles of the medical ethics committee of National Nutrition and Food Technology Research Institute, No.1395.110, and has been registered at the Iranian Center for Clinical Trials (No. IRCT 20100524004010 N22).

### Study participants

Patients with active mild to moderate UC, whose disease had been confirmed pathologically, were recruited for this study. The inclusion criteria included: histopathologic diagnosis of mild to moderate (diagnosis of the severity of the disease was based on physician’s judgment), vitamin D deficiency (< 30 ng/mL), the absence of other diseases, intestinal disorders, known autoimmune diseases, cancer, inflammatory and infectious diseases, not using vitamin D supplements, mineral-multivitamins, omega-3, polyphenolic and antioxidant medications, and not using of anticoagulants such as Heparin and Warfarin, non-steroid anti-inflammatory drugs such as Ibuprofen, Aspirin and Diclofenac, Antihistamines and calcium channel antagonists such as Nifedipine during the past month, age > 18, and no change in the type and dosage of their medicine over the past month. The exclusion criteria included pregnancy or breastfeeding or using contraceptives in women, patient’s unwillingness to continue study protocol, changes in the type and dosage of the drug during the study.

### Interventions

In this study, participants were randomly divided into two groups to receive either a high-dose or a low-dose vitamin D supplement. Patients received the supplements for 12 weeks [14, 25], based on their group assignment. Participants in high dose group, received 2 capsules of 1000 IU vitamin D daily [25]. Patients in the low dose group were given 1 capsule of 1000 IU of vitamin D supplements and 1 capsule of placebo daily, which were apparently similar to each other. Supplement capsules were purchased from Zahravi Company. For blinding the study supplements, the boxes containing the capsules were coded as A, B and C, by a person other than the researchers. All of the patients received a box of A, but according to the fact that patients in the group receiving a high or a low dose of vitamin D supplementation, boxes B or C were given to them, as vitamin D or placebo capsules. Therefore, patients received 2000 IU vitamin D or 1000 IU vitmin D plus a placebo capsule. Patients’ compliance was assessed using capsules count remained in the box at each visit (6th, and 12th week of the intervention).

### Measurements

At first, and 12th week of study, the weight of each patient was measured in light weight clothing, with accuracy of 100 g and height measurement of each patient without shoes was performed by meters mounted on a wall with a 1 cm accuracy. Then the body mass index (BMI) was calculated. After 9–12 h of fasting, 5 ^cc^ blood samples were taken from each patient and their serum was kept in the − 80°^c^ freezer until serum measurement analysis.

IBDQ-9 and SCCAIQ were filled out at the beginning and the end of study. Serum concentrations of TAC, TOS and 25- hydroxyl vitamin D (25-OHD) concentration were measured using ELISA method (ZellBio GmbH, Ulm, Germany) [14, 23, 26, 27].

### Statistical analysis

Data are presented as (mean ± standard deviation) and frequency (percentages) for quantitative and qualitative variables, respectively. Normal distribution of data was evaluated using Kolmogorov-Smirnov test. Chi-Square test was used to compare the qualitative confounding variables of the two groups. To compare the mean of quantitative variables in each group (if their distribution was normal) paired t-test was used for double-measured data and comparison of their mean between two groups was evaluated by Student’s t-test.

## Results

Fifty patients (25 in the high dose group and 25 in the low dose group) took part in our study. A total of 46 patients, including 24 patients in the high dose group (52.2%) and the 22 patients in the low dose group (47.8%) completed the study and 4 patients lost to follow-up; the reasons for the withdrawal of patients from both groups are shown in Fig. 1. There was no significant difference in the dropout rate between two groups (*P*-value = 0.609). In general, the participation rate in this study was 92%.

As shown in Table 1, there was no significant difference in the distribution of sex as well as age and length of the disease between high dose and low dose group at the beginning of the study (Table 1). Comparison of BMI and medications during the study did not show any significant difference in each group (Table 2). All patients used Masalamine at the beginning and the end of study. Forty one percent of participants in low dose group and 16.6% in high dose group took Azathioprine (*p* = 0.55). Corticosteroids were used by 12.5, and 13.6% of patients in high dose and low dose groups, respectively (*p* = 1). Moreover, there was no significant difference between the two groups in terms of calorie intake and dietary components.

**Table 1:** The duration of the disease and individual characteristics of the patients participated in two groups receiving high and low doses of vitamin D before intervention *

Variables	Low dose group *N* = 22	High dose group *N* = 24	*P*-value
Gender (n(%))			1^a^
Men	11 (50)	13 (54.2)	
Women	11 (50)	11 (45.8)	
Age (years)	39.72 ± 15.56	34 ± 12.48	0.174^b^
Men	35.00 ± 14.89	33.31 ± 14.62	0.782^b**^
Women	44.45 ± 15.41	34.82 ± 10.02	0.098^b**^
Duration of disease (years)	7.18 ± 1.15	4.04 ± 0.97	0.083^b^
Men	6.18 ± 2.18	4.07 ± 1.39	0.408^b**^
Women	8.18 ± 2.15	4.01 ± 1.42	0.122^b**^
Extension of Disease (n (%))			0.97^a^
Proctitis	7 (32)	7 (30)	
Left side	14 (63)	16 (66)	
Pancolitis	1 (4)	1 (4)	

*The values for age and duration of the disease are reported as mean ± SD and the others reported as number (percentages)

^**^Variables test between the two groups separated by gender

^a^Fisher’s Exact Test

^b^Student’s t-test

**Table 2:** Body mass index and medications in patients participated in two groups receiving high and low doses of vitamin D in the beginning and the end of the study *

Variables	Time of the study		*P*-value^a^
	Beginning of study	End of study	
Body mass index (kg/m^2^)			
High dose group	24.29 ± 3.61	24.69 ± 3.34	0.087
Low dose group	25.56 ± 4.27	25.69 ± 4.39	0.192
*P*-value^b^	0.284	0.382	
Dose of Azathioprine (mg/day)			
High dose group	87.50 ± 25	87.50 ± 25	1
Low dose group	83.33 ± 35.35	83.33 ± 35.55	1
*P*-value^b^	0.837	0.837	
Dose of Corticosteroid (mg/day)			
High dose group	2.5 ± 7.3	0.9 ± 2.5	0.16
Low dose group	0.2 ± 1.0	0	0.32
*P*-value^b^	0.16	0.10	
Dose of Mesalamine (mg/day)			
High dose group	2473.68 ± 1123.90	2450 ± 1099.04	0.331
Low dose group	2523.81 ± 872.87	2523.81 ± 872.87	0.696
*P*-value^b^	0.875	0.813	

* Values are reported as Mean ± standard deviation

^a^within groups; ^b^ between groups

Serum 25-OHD level was not significantly different between two groups at the beginning (*p* = 0.37), and the end of study (*p* = 0.93). In the high dose group, serum 25-OHD significantly increased during the study, while this increase was not significant in the low dose group (Table 3). The mean changes in low dose and high dose groups were 0.2 ± 2.2 and 6.7 ± 3.8 respectively, which was significantly higher in high dose group compared with low dose group (*p* < 0.0001).

**Table 3:** Mean and standard deviation of vitamin D levels in patients participated in two groups taking high and low doses of vitamin D at the beginning and end of the study*

Variables	Time of the study		*P*-value^a^
	Beginning of study	End of study	
Serum 25-hydroxy vitamin D (ng/mL)			
High dose group	21.83 ± 9.69	28.99 ± 8.69	< 0.001
Low dose group	24.37 ± 8.14	28.75 ± 11.90	0.192
*P*-value^b^	0.377	0.936	

* Values are reported as Mean ± standard deviation

^a^within groups; ^b^ between groups

Serum concentrations of TOS and TAC, oxidant/antioxidant concentrations did not change significantly in any of the two groups during the study.

In the high dose group, serum TOS concentration increased significantly (*P* = 0.023) compared to the low dose group; however, this significance was eliminated after adjusting for confounders (*P* = 0.514). The change in serum TAC concentration between two groups during the study was not statistically significant and remained unchanged after adjusting for confounders (Table 4).

**Table 4:** Mean and standard deviation of serum total oxidative and anti-oxidative capacity in patients participated in two groups taking high and low doses of vitamin Dat the beginning and end of the study

Variables	Time of the study				
	Beginning of study	End of study	*P*-value^c^	^a^*P*-value	^b^*P*-value
Serum TOS (ng/mL)				0.023	0.514
Low dose group	2.94 ± 1.05	3.03 ± 0.80	0.70		
High dose group	3.37 ± 0.96	2.99 ± 1.00	0.17		
*P*-value ^d^	0.15	0.90			
Serum TAC (pg/mL)				0.209	0.599
Low dose group	0.57 ± 0.11	0.56 ± 0.12	0.47		
High dose group	0.57 ± 0.13	0.56 ± 0.09	0.30		
*P*-value^d^	0.93	0.86			

The values are reported as mean ± standard deviation

^a^*P* –value for complementary efficacy (changes comparison) by Student T-test

^b^*P*-value for complementary efficacy (changes comparison) after adjustment by ANCOVA test for BMI, and baseline values

^c^*P* –value for within group comparison using paired t test

^d^*P* –value for between groups comparison using Student T-test

As shown in Table 5, the mean score of IBDQ-9 after vitamin D supplementation significantly increased in both groups compared to baseline value. In the high dose group, the IBDQ-9 mean score showed a significant increase compared to the low dose group (*P* value = 0.001), which remained significant after adjusting for confounding factors (*p* = 0.003). The SCCAI score in both high dose and low dose groups was reduced at the end of the study compared to the beginning of the study (*P* = 0.009, and *p* ≥ 0.001, respectively). In the high dose group, the SCCAI mean scores in comparison with the low dose group showed a significant decrease (*P* = 0.004), which remained significant after adjustment for confounders (*p* = 0.045).

**Table 5:** Mean and standard deviation score of quality of life and clinical activity score in patients participated in two groups taking high and low doses of vitamin D at the beginning and end of the study

Variables	Time of the study				
	Beginning of study	End of study	*P*-value^c^	^a^*P*-value	^b^*P*-value
Quality of life questionnaire score				0.001	0.003
Low dose group	42.59 ± 8.66	44.73 ± 8.01	< 0.001		
High dose group	40.54 ± 9.46	46.75 ± 9.27	< 0.001		
*P*-value^d^	0.45	0.43			
Clinical Activity Indicator Questionnaire score				0.004	0.045
Low dose group	3.00 ± 3.59	2.68 ± 2.27	0.009		
High dose group	5.25 ± 2.98	2.67 ± 2.25	< 0.001		
*P*-value^d^	0.06	0.98			

The values are reported as mean ± standard deviation

^a^*P* –value for complementary efficacy (changes comparison) by Student T-test

^b^*P*-value for complementary efficacy (changes comparison) after adjustment by ANCOVA test for BMI, and baseline values

^c^*P* –value for within group comparison using paired t test

^d^*P* –value for between groups comparison using Student T-test

## Discussion

To our knowledge, this study is the first double blind randomized clinical trial in adult patients with UC, which has assessed the effects of two dosages of vitamin D supplementation on its serum concentration, disease activity index, quality of life and body oxidative stress status. Our results have shown that daily dose of 2000 IU vitamin D increases 25-OHD level significantly; however, this result in daily dose of 1000 IU was not observed. Based on the recommended dietary allowance (RDA), the daily requirement dosage of vitamin D in adults (> 18 years) is 600 IU [28] but a recommendation for supplementing vitamin D in IBD patients is not available. Some studies have shown that vitamin D bioavailability decreases in patients with IBD; however, it is completely variable in different patients [29, 30]. In addition to vitamin D malabsorption, inadequate exposure to sunlight either related to lifestyle or persistent symptoms of active disease restricting physical activity, inadequate dietary intake due to symptoms of IBD, impaired conversion of vitamin D to its active products, increased catabolism and excretion are among high vitamin D deficiency prevalence in patients with IBD [31, 32].

Regarding malabsorption experienced by UC patients, we considered a higher level of vitamin D (2000 IU) as their daily required dosage [33]. Also, to provide the low dose group sufficient amount of vitamin D, they were given a daily dose of 1000 IU.

Limited studies have evaluated the effects of vitamin D supplementation on its serum concentration in pediatric patients with IBD. Simek et al [34] evaluated the effects of two dosages of 5000 IU of vitamin D3 per 10 kg of body weight per week and 10,000 IU of vitamin D3 per 10 kg of body weight per week for a total of 6 weeks in pediatric IBD patients. The concentration of this vitamin in both groups increased compared to the beginning of the study at weeks 8 and 12.Papa et al. [25] evaluated the effects of daily dose of 2000 IU vitamin D2(control), and daily dose of 2000 IU vitamin D3 and weekly dose of 50′000 IU vitamin D2 in pediatric patients with IBD. Their results showed that oral doses of 2000 IU vitamin D3 daily and 50,000 IU of vitamin D3 per week were superior to daily dose of 2000 IU vitamin D2 and were better tolerated in children and adolescents with IBD.

Moreover, our results showed that both doses of vitamin D improved patients’ quality of life; however, level of disease activity only reduced by daily dose of 2000 IU. Although no clinical trial has reported the effects of vitamin D on disease activity and patients’ quality of life, the results of cross-sectional studies showed that the clinical activity and quality of life of IBD patients had a significant relationship with lower levels of vitamin D [20, 35]. A South African cohort study on Crohn’s disease found a relation between low levels of vitamin D and increased activity of the disease [19]. In two other studies, it has been shown that low levels of vitamin D are common in IBD patients, which has been associated with mortality and severity of the disease as well as the early onset of it, which could indicate the importance of the role of this vitamin in the improvement of these patients [19, 22]. Another study showed that levels of 35 ng/mL or less of serum vitamin D during the treatment period would increase the risk of recurrence of UC [21].

Although previous studies have shown that high levels of vitamin D is associated with low frequency of relapses [36], it is not known that blood vitamin D affects the disease relapse, or disease activity affects vitamin D status?

Vitamin D deficiency is associated with various immunological diseases, such as allergies and autoimmune diseases. Different mechanisms for the effects of vitamin D on the inherent and acquired immune system are intended to reduce inflammation, promote immune tolerance, and increase the integrity of the intestinal epithelium [16]. Vitamin D is involved in the regulation of the immune system and may play a pivotal role in the pathogenesis of IBD and it is considered as a contributing factor in the treatment of IBD [25].

Our results showed that daily dose of vitamin D does not have any significant effect on oxidative stress status. Vitamin D plays an important role in a wide range of physiological functions including immune responses [15]. Vitamin D inhibits several pro-inflammatory pathways [37, 38], modifies autophagy [38], reduces the oxidative stress [39], the differentiation and activation of the white blood cells [38, 40, 41] and increases the expression of tight junctions in the intestinal epithelium, thereby affecting mucosal permeability and tissue integrity [42].

Based on experimental studies, vitamin D receptor (VDR) and its ligands have an important effect on IBD disease [43]. Cantorna et al. [44] reported that mice with both vitamin D and IL-10 deficiency showed more acute entero-colitis at their 7th week of life. These authors reported that VDR plays an important role in capacity of colonic epithelium healing.

Wang et al. [45] showed that vitamin D deficiency exacerbates oxidative stress in obese patients. Vitamin D plays an anti-oxidative role through the regulation of oxidative stress reducing proteins [46, 47]. In this study, the primary level of oxidative stress was low, and this might be the reason of not observing a significant effect of vitamin D on oxidant and antioxidant concentrations.

The strength of the current study is evaluation of the effects of two doses of vitamin D supplements on serum 25-OHD levels, oxidative factors, quality of life, and disease activity index in adult patients with active mild to moderate UC as the first double-blind randomized clinical trial.

Our study has some limitations. It may be necessary to use vitamin D for a longer time or in higher doses in order to observe its effects on oxidative stress status. Another limitation of our study was the lack of a healthy control group to compare the results with the healthy individuals. Moreover, we did not measure systemic phlogosis markers in this study; however, we assessed the oxidative stress status and disease activity which are correlated with systematic inflammation.

## Conclusion

In conclusion, this study revealed that vitamin D supplementation with 2000 IU / day for 12 weeks could lead to an increase in serum 25-OHD levels, improvement of quality of life, and decrease in the disease activity index in adult patients with active mild to moderate UC, while supplementation with vitamin D of 1000 IU / day for 12 weeks, only improved the quality of life of patients. In both groups, the serum levels of TAC and TOS patients were not affected significantly.

## Abbreviations

25(OH)2D: 25-hydroxyvitamin D
BMI: Body mass index (Kg/m2)
CD: Crohn’s disease
IBD: Inflammatory bowel disease
IBDQ-9: Inflammatory bowel disease questionnaire-9
IU: International units
SCCAIQ: Simple Clinical Colitis Activity Index Questionnaire
TAC: Total anti-oxidant status
TOS: Total oxidant status
UC: Ulcerative colitis
